# Collective Value Promotes the Willingness to Share Provaccination Messages on Social Media in China: Randomized Controlled Trial

**DOI:** 10.2196/35744

**Published:** 2022-10-04

**Authors:** Chunye Fu, Xiaokang Lyu, Mingdi Mi

**Affiliations:** 1 Department of Social Psychology Zhou Enlai School of Government Nankai University Tianjin China; 2 Students' Affairs Division Weinan Vocational ＆ Technical College Weinan China

**Keywords:** individual value, collective value, vaccination, message-sharing willingness, perceived responsibility, misinformation, vaccine misinformation, public health, influenza vaccine, social media, COVID-19

## Abstract

**Background:**

The proliferation of vaccine misinformation on social media has seriously corrupted the public’s confidence in vaccination. Proactively sharing provaccination messages on social media is a cost-effective way to enhance global vaccination rates and resist vaccine misinformation. However, few strategies for encouraging the public to proactively share vaccine-related knowledge on social media have been developed.

**Objective:**

This research examines the effect of value type (individual vs collective) and message framing (gain vs loss) on influenza vaccination intention (experiment 1) and the willingness to share provaccination messages (experiment 2) among Chinese adults during the COVID-19 pandemic. The primary aim was to evaluate whether messages that emphasized collective value were more effective in increasing the willingness to share than messages that emphasized individual value.

**Methods:**

We enrolled 450 Chinese adults for experiment 1 (n=250, 55.6%) and experiment 2 (n=200, 44.4%). Participants were randomly assigned to individual-gain, individual-loss, collective-gain, or collective-loss conditions with regard to the message in each experiment using the online survey platform’s randomization function. Experiment 1 also included a control group. The primary outcome was influenza vaccination intention in experiment 1 and the willingness to share provaccination messages in experiment 2.

**Results:**

The valid sample included 213 adults in experiment 1 (females: n=151, 70.9%; mean age 29 [SD 9] years; at least some college education: n=202, 94.8%; single: n=131, 61.5%) and 171 adults in experiment 2 (females: n=106, 62.0%; mean age 28 [SD 7] years; at least some college education: n=163, 95.3%; single: n=95, 55.6%). Influenza vaccination intention was stronger in the individual-value conditions than in the collective-value conditions (*F*_3,166_=4.96, *P*=.03, *η*^2^=0.03). The reverse result was found for the willingness to share provaccination messages (*F*_3,165_=6.87, *P*=.01, *η*^2^=0.04). Specifically, participants who received a message emphasizing collective value had a higher intention to share the message than participants who read a message emphasizing individual value (*F*_3,165_=6.87, *P*=.01, *η*^2^=0.04), and the perceived responsibility for message sharing played a mediating role (indirect effect=0.23, 95% lower limit confidence interval [LLCI] 0.41, 95% upper limit confidence interval [ULCI] 0.07). In addition, gain framing facilitated influenza vaccination intention more than loss framing (*F*_3,166_=5.96, *P*=.02, *η*^2^=0.04). However, experiment 2 did not find that message framing affected message-sharing willingness. Neither experiment found an interaction between value type and message framing.

**Conclusions:**

Strengthened individual value rather than collective value is more likely to persuade Chinese adults to vaccinate. However, these adults are more likely to share a message that emphasizes collective rather than individual value, and the perceived responsibility for message sharing plays a mediating role.

## Introduction

### Background

The proliferation of vaccine misinformation on social media has seriously corrupted the public’s confidence in vaccination [1-3]. Extreme antivaxers create vaccine misinformation online [4-6], and ordinary users often spread vaccine misinformation voluntarily on social media [7,8] because of the emotional and attractive nature of misinformation. Fortunately, social media platforms are increasingly implementing positive interventions to resist vaccination misinformation [9,10].

These endeavors mainly focus on 2 different approaches. The first approach relies on information technology, such as fact-checking labels [9] or debiasing strategies [11], to reduce the impact of existing vaccine misinformation on its audience. The other approach provides more vaccination knowledge on social media to suppress the diffusion of vaccine misinformation [4,8,12,13].

However, additional strategies to mobilize the public to debunk vaccine misinformation on social media are needed to successfully achieve public health goals. Just as vaccine misinformation can drive sharing intentions, provaccination information, if presented properly, can motivate the public’s willingness to share on social media. Inspiring the public’s willingness to share provaccination information can improve the current situation in which antivaccination messages occupy a more controversial space than provaccination messages on social media [8,14], and prevent the adverse effects of misinformation [15]. Hence, the main goal of this study is to explore strategies to present effective messages to improve the public’s willingness to share provaccination messages on social media.

Previous studies have confirmed that emphasizing the collective benefits of vaccination, such as the long-term benefits of herd immunity for society, could positively affect participants’ vaccination intention [8,16,17]. In particular, according to the “sharing for the community” model [18], community interest serves as a more influential motivation for the online sharing of health-related knowledge than personal gain does [19], and information-sharing behaviors driven by community interest are based on reciprocity norms [20]. Therefore, it is logical to hypothesize that provaccination messages that emphasize collective value can promote the willingness to share provaccination messages better than those that emphasize individual value.

In addition, message framing plays a key role in health communication [21]. Based on gain versus loss message framing [22,23], the value of vaccination can be framed by highlighting either desirable consequences (eg, health benefits) or undesirable consequences (eg, disease risk). Although a meta-analytic review showed that gain-framing messages are significantly more likely to encourage illness prevention behaviors compared to loss-framing messages [22], another study showed that framing effects are significant only when individuals perceive the issue to have high personal relevance [24]. Therefore, we assume that the framing effects may be less obvious when messages are described with collective value, since personal relevance is diminished when messages are described with collective value compared to individual value. Thus, we used message framing as an independent variable to explore whether message framing and value type synergistically affect vaccination message-sharing intention.

### Goal of This Study

We conducted 2 survey experiments during the COVID-19 pandemic outbreak in mainland China. Experiment 1 explored whether a message that emphasized collective value improved vaccination intention more than one that emphasized individual value during the COVID-19 pandemic outbreak. Experiment 2 tested our primary hypothesis of whether collective value significantly facilitates the dissemination of provaccination messages online. We also measured the perceived importance and responsibility of message sharing as mediating variables in experiment 2 to examine the mechanism of collective value on the willingness to share messages.

## Methods

### Design

Two 2 (value type: collective vs individual) × 2 (message framing: gain vs loss) between-participant experiments were conducted using Sojump [25], an online study portal. Experiment 1 explored the main effect of value type and message framing on vaccination intention for the influenza vaccine (including a no-message control group), while experiment 2 focused on message-sharing willingness.

### Participants

The sample size was estimated using G*Power 3.1 [26], assuming a statistical power of 90%, a significance level of .05, and an effect size of 0.25. The estimated minimum sample size for 2-factor ANOVA was 172 (n=43, 25%, for each condition). Accordingly, we recruited 450 volunteers in total (experiment 1: n=250, 55.6%; experiment 2: n=200, 44.4%) for monetary compensation.

All participants were native Chinese readers; none had a medical education background or were from Wuhan, China. Manipulation checks in the survey resulted in a total of 384 valid participants in the experiments (experiment 1: n=213, 85.2%; experiment 2: n=171, 85.5%). Table 1 presents the descriptive information about the participants.

**Table 1:** Descriptive information about participants in experiments 1 and 2.

Variables		Experiment 1 (N=213)	Experiment 2 (N=171)
**Age (years)**			
	Range	18-58	18-56
	Mean (SD)	29 (9)	28 (7)
**Gender, n (%)**			
	Female	151 (70.9)	106 (62.0)
	Male	62 (20.1)	65 (38.0)
**Marital status, n (%)**			
	Single	131 (61.5)	95 (55.6)
	Married	82 (38.5)	76 (44.4)
**Education, n (%)**			
	Middle school graduate and below	2 (0.9)	2 (1.2)
	High school graduate	9 (4.2)	6 (3.5)
	College degree holder	129 (60.6)	124 (72.5)
	Graduate degree holder and above	73 (34.3)	39 (22.8)

### Message Stimuli and Pretest

#### Message Stimuli

The content of the messages was developed based on considerable scientific research published in vaccination-related fields [8,27,28]. We wrote 5 consecutive paragraphs, with an associated theme in each message. In approximately 450 Chinese characters, each of the messages described a story about immunization and stated the substantial value of vaccination. To increase the vividness of the messages, we used pictures from a royalty-free image web archive.

**Figure 1:**
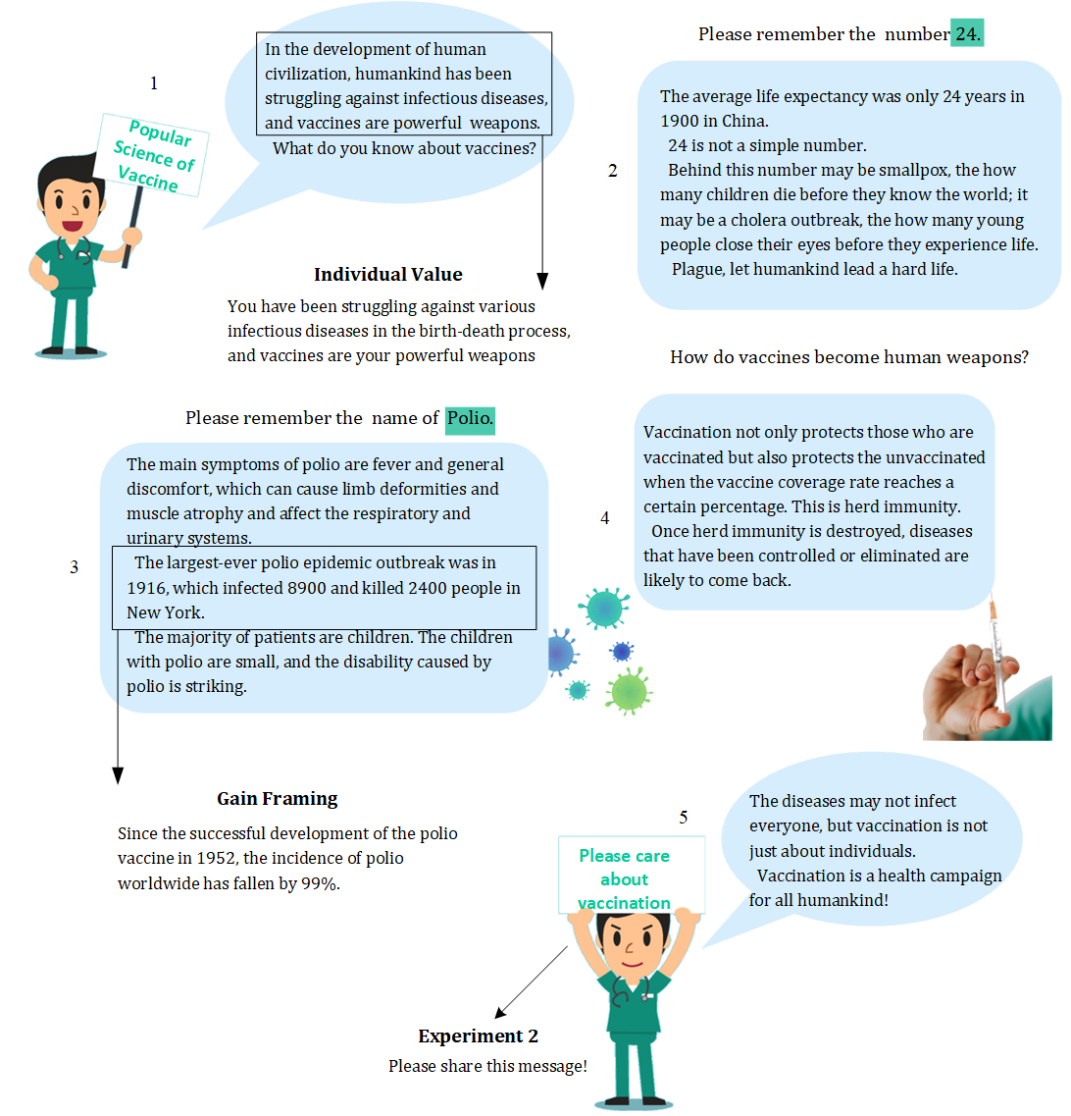
Material for the collective-loss condition for experiment 1.

Figure 1 presents the condition of collective loss in experiment 1. Value type was manipulated by emphasizing collective or individual value under different conditions. For example, herd immunity was explained in the condition of collective value, while individual immunity was used in the condition of individual value. In addition, the subjects of the sentences were changed from “human” (collective value) to “you” (individual value).

In line with prior framing-related literature [29], message framing was described separately by strengthening the benefit of vaccination in gain framing or the harm of not being vaccinated in loss framing. We also used specific examples of vaccination to convey the messages (eg, the polio vaccine has saved human lives, or polio has killed humans).

#### Pretest of Messages

We tested the validity of the messages with 120 participants (the manipulation check resulted in 111 valid participants) who did not participate in the formal experiments. The 111 participants (females: n=83, 74.8%; mean age 33 [SD 12] years; at least some college education: n=93, 83.7%; single: n=58, 52.3%) were native Chinese readers; none had a medical education background or were from Wuhan, China. Each participant was randomly assigned to 1 of the 4 materials using the online survey platform’s randomization function.

First, we used a single 7-point item to measure participants’ credibility ratings of the messages. The responses showed no significant differences between the credibility ratings for collective and individual value (*F*_3,107_=1.45, *P*=.23) or loss and gain framing (*F*_3,107_=0.003, *P*=.95). Second, we asked participants to choose the message they read with regard to collective or individual value describing the benefits of vaccination or the risk of not vaccinating. The results revealed that more than 90% of the participants could accurately distinguish collective or individual value and gain or loss framing. Thus, the materials were considered valid for use in subsequent experiments.

### Procedure

**Figure 2:**
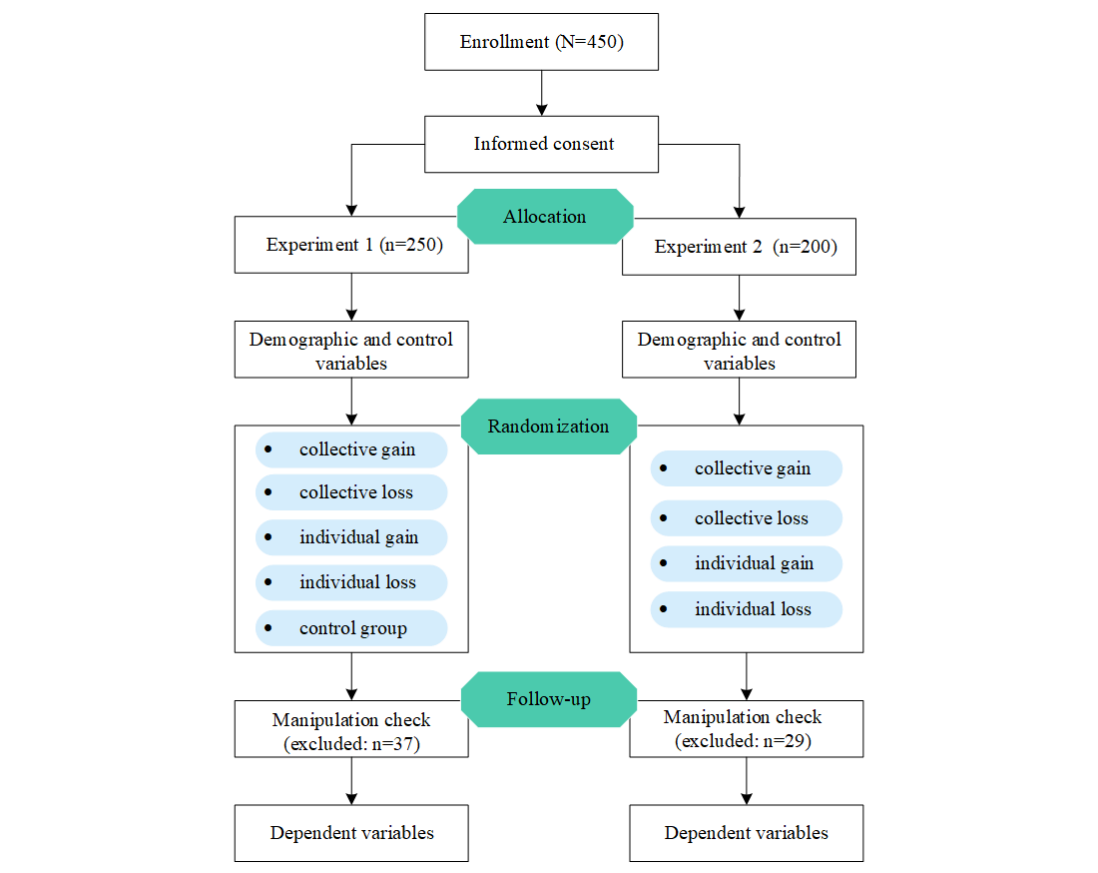
Schematic representation of the experimental flow.

The system flowchart can be seen in Figure 2. After obtaining informed consent, the survey gathered information about the participants’ demographics (gender, age, education, and marital status) and control variables. The participants were randomly assigned to 1 of the conditions using the online survey platform’s randomization function. Those assigned to 1 of the 4 message conditions proceeded to a page that showed them a message and required them to answer 2 manipulation check questions. They were then instructed to respond to questions related to the variables described in the following section. Those in the control condition in experiment 1 proceeded directly to a similar survey without reading a message.

### Measures

All control and dependent variables used in our experiments are presented in Table 2. The details of all measures used in experiments are provided in Multimedia Appendix 1.

**Table 2:** Control and dependent variables used in experiments 1 and 2.

Variables	Experiment(s)	Measures	Items, n	Sample item	Score range	Cronbach α
Control	1 and 2	Vaccine confidence	3	Overall, I think vaccines are safe.	1=extremely disagree to 5=extremely agree	.85 (experiment 1) and .72 (experiment 2)
Control	2	Propensity to share on social media	3	I share new messages on social media.	1=rarely to 5=always	.72
Dependent	1	Vaccine acceptance	10	Vaccines are effective at preventing diseases.	1=strongly disagree to 7=strongly agree	.76
Dependent	1	Influenza vaccination intention	3	How likely is it that you are going to receive the flu vaccination this winter?	1=extremely unlikely to 5=extremely likely	.82
Dependent	2	Perceived importance of message	3	The message contains crucial knowledge about the vaccine.	1=extremely disagree to 5=extremely agree	.74
Dependent	2	Perceived responsibility for message sharing	6	At this moment, I believe I have the responsibility for sharing this message to improve the influenza vaccination rate.	1=strongly disagree to 7=strongly agree	.89
Dependent	2	Message-sharing willingness	3	I will share this message using different social networking tools.	1=extremely disagree to 5=extremely agree	.72

### Control Variables

Vaccine confidence, assessed by modifying the 4-item vaccine confidence scale to 3 items by removing the item about religion [30], was measured as a control variable in experiments 1 and 2. The propensity to share on social media [31] served as a control variable in experiment 2.

### Dependent Variables

We focused on vaccination intention for the 2020-2021 influenza vaccine. A self-administered scale was used. Specifically, participants were provided with medical news. The news read, “Once COVID-19 infection occurs together with influenza, the difficulty of diagnosis will be increased…” In addition, the intention to receive the influenza vaccination was measured by how likely the participants were to accept the influenza vaccination for themselves or recommend it to their families in the winter. The secondary dependent variable in experiment 1 was vaccine acceptance, which captured the entire conceptual domain of vaccine acceptance [32].

Experiment 2 comprised 3 dependent variables: the perceived importance of messages [33], the perceived responsibility for message sharing [34], and message-sharing willingness [33].

### Ethical Considerations

The study was conducted according to the guidelines of the Declaration of Helsinki and approved by the Institutional Review Board of Nankai University (#NKUIRB2020023, approval date February 25, 2020).

## Results

### Experiment 1

The descriptive and inferential statistics of experiment 1 are shown in Table 3. Demographic variables were also included in the analysis as control variables.

**Table 3:** Means (SDs) for each condition and inferential statistics of each condition compared to the control group of experiment 1.

Value and framing		Sample size (N=213), n (%)	Vaccine acceptance			Vaccination intention	
			Mean (SD)	*P* value	Mean (SD)		*P* value
**Collective**							
	Gain	43 (20.2)	5.46 (0.79)	.02	3.91(0.87)		.01
	Loss	41 (19.2)	5.43 (0.65)	.06	3.57(0.89)		.48
**Individual**							
	Gain	44 (20.7)	5.53 (0.71)	.02	4.11(0.65)		<.001
	Loss	42 (19.7)	5.60 (0.65)	.01	3.94(0.63)		<.001
Control group		43 (20.2)	5.06 (0.74)	N/A^a^	3.35(1.12)		N/A

^a^N/A: not applicable.

#### Vaccine Acceptance

The main effects of value type (*F*_3,166_=0.16, *P*=.69), message framing (*F*_3,166_=0.001, *P*=.97) and 2-way interactions between value type and message framing (*F*_3,166_=0.49, *P*=.49) were not statistically significant for vaccine acceptance. Further pairwise comparisons showed that participants who had received a message on any condition expressed significantly higher vaccine acceptance than those in the control group. Except for the collective-loss condition, the difference was only borderline significant.

#### Influenza Vaccination Intention

The results of the influenza vaccination intention index revealed a significant main effect of value type (*F*_3,166_=4.96, *P*=.03, *η*^2^=0.03). Vaccination intention was stronger in the individual-value conditions than in the collective-value conditions. Messaging framing also exhibited a significant main effect (*F*_3,166_=5.96, *P*=.02, *η*^2^=0.04). Vaccination intention was stronger in the gain-framing condition than in the loss-framing condition. No interaction effects were significant (*F*_3,166_=0.83, *P*=.36). We also found that the participants in all conditions except for the collective-loss condition had significantly higher vaccination intention than those in the control group.

### Experiment 2

The descriptive statistics of experiment 2 are shown in Table 4.

**Table 4:** Means (SDs) for each condition of experiment 2.

Value and framing		Sample size (N=171), n (%)	Perceived importance, mean (SD)	Perceived responsibility, mean (SD)	Message-sharing willingness, mean (SD)
**Collective**					
	Gain	43 (25.1)	4.54 (0.43)	6.19 (0.62)	3.96 (0.62)
	Loss	42 (24.6)	4.61 (0.42)	6.23 (0.67)	4.04 (0.68)
**Individual**					
	Gain	44 (25.7)	4.44 (0.51)	5.87 (0.77)	3.75 (0.81)
	Loss	42 (24.6)	4.21 (0.56)	5.62 (1.07)	3.44 (1.01)

#### Perceived Importance, Perceived Responsibility, and Message-Sharing Willingness

Significant differences were found between collective and individual value. Participants who received a message emphasizing collective value had higher perceived importance (*F*_3,165_=4.72, *P*=.03, *η*^2^=0.03), higher perceived responsibility (*F*_3,165_=9.31, *P*=.003, *η*^2^=0.06), and a higher willingness to share the message (*F*_3,165_=6.87, *P*=.01, *η*^2^=0.04) than participants who read a message emphasizing individual value. There was no significant main effect of message framing or 2-way interactions between value type and message framing on each dependent variable.

**Figure 3:**
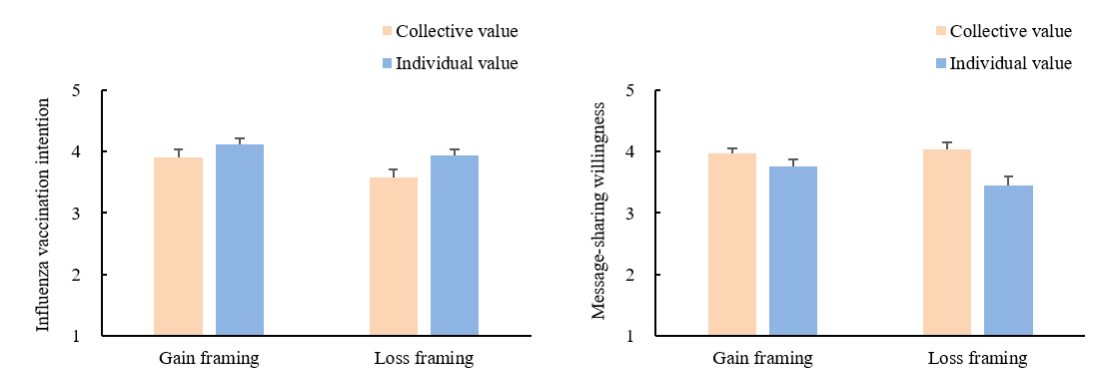
Effect of value type on influenza vaccination intention (experiment 1) and message-sharing willingness (experiment 2).

Influenza vaccination intention and message-sharing willingness were the 2 primary outcomes in experiments 1 and 2, respectively. Therefore, we present the 2 dependent variables together in Figure 3 to facilitate a comparison of trends.

#### Mediating Effect Analysis

A mediating effect analysis was conducted to determine whether the effect of collective value was mediated by perceived importance and responsibility. The mediational model was assessed using the SPSS PROCESS macro (model 4) with a 95% bias-corrected CI based on 5000 bootstrap samples [35]. The value type (collective value=1, individual value=0) was used as the independent variable, perceived importance and responsibility were parallel mediators, and message-sharing intention was the outcome variable.

**Figure 4:**
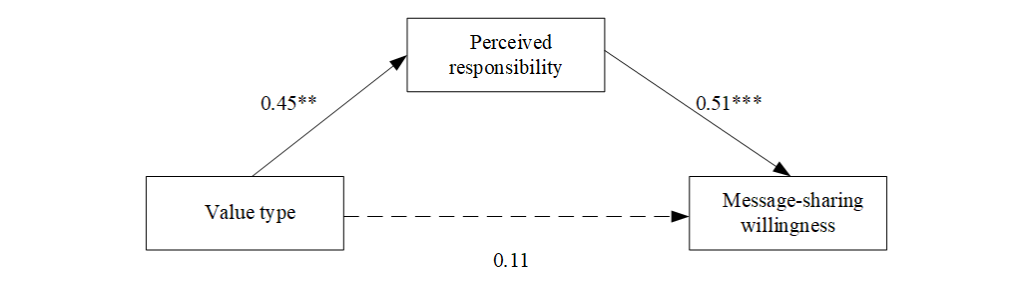
Statistical model of the meditational model. **P<.01; ***P<.001.

The analysis yielded a significant model (*F*_8,162_=6.88, *P*<.001, R^2^=0.25). The direct effect of value type on the intention to share was not significant after introducing the indirect effects of perceived importance and responsibility into the model (Figure 4). One indirect effect of perceived importance was also not significant (indirect effect=0.03, 95% lower limit confidence interval [LLCI] 0.11, 95% upper limit confidence interval [ULCI] −0.01). In contrast, the other indirect effect of perceived responsibility was significant (indirect effect=0.23, 95% LLCI 0.41, 95% ULCI 0.07).

## Discussion

### Principal Findings

In experiment 1, we found that individual value had a more substantial effect than collective value on influenza vaccination intention. Interestingly, the results from experiment 2 indicated that the message describing collective value promoted message-sharing willingness better than the message related to individual value; the perceived responsibility for message sharing mediated the effect of collective value on message-sharing willingness but not on perceived importance.

In general, individual-focused messages increased vaccination intention, but collective-focused messages increased the likelihood of sharing. These results seem to produce conundrums for message designers and reveal that designers should finely construct specific messages for different targets. For example, if the goal is to make provaccination messages occupy more space on social media, then collective value should be prioritized to describe messages. In another circumstance, if the improvement of vaccination rates is more important and urgent, especially under circumstances with high risks, individual value should be considered first. Moreover, the message of collective value described as gain framing also enhanced vaccination intention compared to the control group. Therefore, collective-gain messages effectively increase vaccination intention, while enhancing message-sharing willingness.

This study also obtained some other results. First, there were no differences between collective and individual value or gain and loss framing on vaccine acceptance; however, vaccine acceptance improved in all intervention groups compared to the control group. Second, gain framing was more effective than loss framing in influenza vaccination intention. Finally, we did not find an interaction effect between value type and message framing on any dependent variables.

These results contribute to the literature on online health knowledge sharing and provide implications for practice.

### Limitations

There are some limitations of this study. First, we did not design a condition that combined collective and individual value and that tested whether such messages can promote both vaccination intention and message-sharing willingness. This limitation can be addressed through follow-up research.

Second, the external validity of this study is limited. In a realistic network environment, other types of information with high attractiveness or interesting themes, such as stories about celebrities or sports news, may cause the public to ignore provaccination messages [36]. Therefore, it is necessary to simulate the real network context to verify the robustness of the results of this study.

Third, we conducted the research in the early stages of COVID-19. During this period, the epidemic in Wuhan, China, was more serious, while people in other regions were mostly in a state of unknown risk. As the form of the epidemic continues to change in China, the public’s perception of the risk will also change dynamically. This may further lead to changes in the way messages function in different severe situations. Subsequent studies could consider collecting data at different time points and drawing on studies that use time series models [37,38] in an attempt to optimize health strategies to resist vaccine misinformation and boost vaccination.

Finally, the participants were mainly female, younger, and single and had a college degree or above. Although we controlled for these demographic variables in the analysis, the external validity of the experimental results is limited. This study can be further improved to examine whether the function of the messages varies among populations.

Despite the limitations, the study complements and extends previous studies that focused on collective benefits in personal vaccination decisions.

### Comparison With Prior Work

The effect of value type on vaccination intention contradicts a previous study that found that collective benefit promotes vaccination intention more than individual benefit does [16]. Notably, a study supports our finding, showing that individuals self-categorized as being in a high-risk group are more likely to adopt vaccination behavior following self-benefit messages than social benefit messages [39]. Our study was conducted during the COVID-19 outbreak, and high risk perception might have resulted in the public’s heightened sensitivity to individual value messages.

In contrast, message-sharing willingness was promoted by collective value more than individual value, and perceived responsibility played a mediatory role. These findings are consistent with a similar study that found that social integrative benefits significantly and positively influence knowledge-sharing intention in virtual communities [40].

Finally, the results of message framing coincide with a previous study that suggested that gain-framing messages are more likely to encourage illness prevention behaviors compared to loss-framing messages [22].

### Conclusion

Online messages emphasizing individual value promote the intention to vaccinate more strongly than those emphasizing collective value, but this effect is reversed in message-sharing willingness. Furthermore, perceived responsibility for message sharing plays a mediating role between collective value and message-sharing willingness. Our findings have practical implications for constructing and providing effective, targeted provaccination messages.

## Appendix

Multimedia Appendix 1 Details of all measures used in experiments.

Multimedia Appendix 2 CONSORT checklist.

## Abbreviations

LLCI: lower limit confidence interval
ULCI: upper limit confidence interval
