# Supplementary material for: Collective Value Promotes the Willingness to Share Provaccination Messages on Social Media in China: Randomized Controlled Trial
Source: JMIR Form Res. 2022 Oct 4;6(10):e35744. doi: 10.2196/35744 (PMC9534273; doi:10.2196/35744)
Supplement: Multimedia Appendix 1 [file formative_v6i10e35744_app1.docx]

| Measures | Items |
| --- | --- |
| **Vaccine confidence**  1 = extremely disagree;  5 = extremely agree | 1. Vaccines are important for children to have.  2. Overall I think vaccines are safe.  3. Overall I think vaccines are effective. |
| **Propensity to share on social media**  1 = rarely  5 = always | 1. I share new messages on social media.  2. I respond to others' messages on social media.  3. I share my experience with others on social media. |
| **Vaccine acceptance**  1 = strongly disagree  7 = strongly agree | 1. Vaccines are safe.  2. Vaccines contain dangerous ingredients.  3. Some vaccines are unnecessary since they target  relatively harmless diseases.  4. Vaccines are effective at preventing diseases.  5. We give children the right number of vaccines.  6. We give children too many vaccines.  7. Vaccines conflict with my belief that children should  use natural products and avoid toxins.  8. Vaccines are a major advancement for humanity.  9. The government should not force children to get  vaccinated to attend school.  10. To protect public health, we should follow  government guidelines about vaccines. |
| **Influenza vaccination intention**  1 = extremely unlikely  5 = extremely likely | 1. How likely is it that you are going to have the flu vaccination this winter?  2. How likely is it that you are going to recommend the flu vaccination to your families this winter?  3. How likely is it that you are going to pay a higher price to receive the influenza vaccination at another specialized institution in the event of inadequate affordable influenza vaccines at the community hospitals around your residence? |
| **Perceived importance of message**  1 = extremely disagree  5 = extremely agree | 1. The message is important.  2. The message contains crucial knowledge about the vaccine.  3. The message is valuable to me |
| **Perceived responsibility for message sharing**  1 = strongly disagree  7 = strongly agree | 1. At this moment, I feel that sharing this message will play a positive effect on the health of the whole society.  2. At this moment, I feel that sharing this message can bring potential benefits to the health of the whole society.  3. At this moment, I think that the behaviour of sharing this message can affect the health of the whole society.  4. At this moment, I feel that I should take responsibility for the health of the whole society.  5. At this moment, I would like to share this message for the health of the whole society.  6. At this moment, I believe I have the responsibility for sharing this message to improve the influenza vaccination rates. |
| **Message-sharing willingness**  1 = extremely disagree  5 = extremely agree | 1. I will retweet the message.  2. I will share information using different social networking tools.  3. I will share the information with people I care  about |
